# Prevalence and impact of malnutrition on outcomes and mortality of under-five years children with pneumonia: a study from Upper Egypt

**DOI:** 10.1007/s00431-023-05138-2

**Published:** 2023-08-05

**Authors:** Yasser Gamal, Asmaa O. Mahmoud, Sherif A. A. Mohamed, Jaafar I. Mohamed, Yasser F. Abdel Raheem

**Affiliations:** 1 Department of Pediatrics, Faculty of Medicine, Assiut University, Assiut, 71516 Egypt; 2 Department of Chest Diseases and Tuberculosis, Faculty of Medicine, Assiut University, Assiut, 71516 Egypt

**Keywords:** Pneumonia, Malnutrition, Outcomes, Children, Under five, Mortality, Clinical, Upper Egypt

## Abstract

Malnutrition has adverse impacts on under-five children with pneumonia. The purpose of this study was to address the prevalence and impact of malnutrition on under-five years children with pneumonia, admitted to a tertiary large children hospital in Upper Egypt. This study is a prospective case-control study. All under-five children diagnosed with pneumonia who were admitted to Assiut University Children’s Hospital (AUCH) from January 1^st^ to December 31st, 2021, were enrolled. Based on their nutritional assessment, the studied participants were classified into 2 groups: (1): Children with pneumonia and with nutritional deficiency considered as cases, and (2): Children with pneumonia and without nutritional deficiency considered as controls. Three hundred-fifty cases and 154 control subjects were enrolled, respectively. 93.4%, 31.1%, and 61.7% of the cases had underweight, stunting, and wasting, respectively. Among those cases, there were significant differences between survivors and non-survivors with regard to some clinicodemographic factors, laboratory parameters, and anthropometric parameters. Lack of compulsory vaccination, presence of sepsis, and blood transfusion (OR 2.874, 95% CI 0.048 – 2.988, p = 0.004, 2.627, 0.040 – 2.677, p = 0.009, and 4.108, 0.134 – 3.381, p < 0.001, respectively) were significant independent predictors for mortality among malnourished children with pneumonia.

*Conclusion*: Malnutrition has a high prevalence in under-five children with pneumonia in our locality. It has adverse effects on the outcomes and in-hospital mortality of those children. Lack of compulsory vaccination, presence of sepsis, and blood transfusion were significant independent predictors of mortality in malnourished children with pneumonia. Larger multicenter studies are warranted.

**Table:** 

**What is Known:** *• Malnutrition has adverse impacts on under-five children with pneumonia.* *• Malnutrition could be a reason for in-hospital mortality among under-five children with pneumonia.*	
**What is New:** *• Malnutrition has a high prevalence in under-five children with pneumonia in Upper Egypt, with its adverse effects on the outcomes and mortality of those children.* *• Lack of vaccination, presence of sepsis, and blood transfusion are significant independent predictors of mortality in malnourished children with pneumonia in Upper Egypt.*

**What is Known:**

*• Malnutrition has adverse impacts on under-five children with pneumonia.*

*• Malnutrition could be a reason for in-hospital mortality among under-five children with pneumonia.*

**What is New:**

*• Malnutrition has a high prevalence in under-five children with pneumonia in Upper Egypt, with its adverse effects on the outcomes and mortality of those children.*

*• Lack of vaccination, presence of sepsis, and blood transfusion are significant independent predictors of mortality in malnourished children with pneumonia in Upper Egypt.*

## Introduction

Pneumonia accounts for 14% of all deaths of children under 5 years old, killing 740 180 children in 2019 [1]. Underweight, inadequate breastfeeding, lack of immunization, and indoor and outdoor air pollution are identified as risk factors for childhood pneumonia [2].

Malnutrition, which commonly affects infants and young children under 5 years, is the most important underlying risk factor for childhood death and one of the most serious health problems in developing countries [3].

According to the WHO, the term malnutrition refers to two distinct groups of conditions. The first is undernutrition, which includes being underweight (low weight for age), stunting (being short for age), wasting (being underweight for height), and nutritional deficiencies or inadequacies such as lack of essential vitamins and minerals. The second aspect refers to individuals being either overweight or obese [4, 5]. As per the UNICEF/WHO/World Bank Group estimates (2021), 22.3%, 17.8%, and 9.5% of children in Egypt had stunting, overweight, and wasting, respectively [6]. The prevalence and impacts of malnutrition in pediatric pneumonia were shown in many studies before [7–11].

Globally, moderately underweight Children hospitalised with pneumonia are twice as likely to die, and severely underweight children are four and a half times as likely to die compared with children with a normal weight. An estimated two in five children admitted to hospital with pneumonia in low-income and middle-income countries are moderately or severely underweight [12].

However, few Egyptian studies had addressed the prevalence and impact of malnutrition on children with pneumonia [13]. In a recent Egyptian study, malnutrition was present in 12.4% of all children with upper and lower RTIs. Lower RTI and malnutrition were substantially more prevalent among children aged under 2 years (p = 0.048 and p < 0.001, respectively) [13].

Identifying the risk factors for pneumonia, the prevalence, and the impact of malnutrition on pneumonia in children may help healthcare workers to take the essential protective and preventive measures to reduce the burden of such problems, particularly in resource-limited countries.

Therefore, in the current study, we aimed to address the prevalence and impact of malnutrition on under-five years children with pneumonia, admitted to a tertiary large children hospital in Upper Egypt.

## Materials and methods

### Study area

Egypt is the most populous country in North Africa and the fourth-most populous on the African continent. Geographically, Upper Egypt consists of the narrow strip of land stretching from the southern border of Egypt near Sudan to approximately Cairo, where it meets Lower Egypt. Egypt has a population of 104,000,000 (July 8, 2013), of them 36,927,447 (% 35.5) were in Upper Egypt [14]. Assiut governorate lies in the heart of Upper Egypt and has a population of 5,063,598. As of July 2014, children in the age groups 0–4 and 5–9 years represented 11.3% and 10.5% of the total Egyptian population, respectively [14].

Assiut University Children’s Hospital (AUCH) is a 503 beds tertiary children’s hospital that serves pediatric patients all over Upper Egypt. From July 2022 to April 2023, 65,464 and 21,419 patients were seen at the outpatient clinics and Emergency Department, respectively. Eleven thousand seven hundred fifty-one patients were hospitalized at different hospital departments [15].

### Study design and setting

The current study is a hospital-based prospective case-control study. Inclusion criteria included all children diagnosed with pneumonia (aged more than 28 days and till the age of 5 years) who were admitted to AUCH from January 1^st^ to December 31st, 2021. Exclusion criteria included children with congenital cardiac lesions, those with immune deficiency disorders, and those with foreign body (FB) inhalation, and pneumonia due to COVID-19. Pneumonia was defined radiologically as the presence of end-point consolidation or other (non-end-point) infiltrate in the lungs according to the World Health Organization (WHO) radiological classification of pneumonia [16].

### Assessments

Enrolled patients were subjected to thorough clinical, laboratory, and radiological examinations. A history of suspected risk factors for the development of nutritional deficiency was taken (type of feeding, birth weight, compulsory vaccination schedule, living in crowding condition, passive smoking, presence of comorbidities, and history of previous hospital admission). The following anthropometric measures: body height and weight, body mass index (BMI), and head circumference, were taken during hospital admission. The study subjects underwent the initial (on admission) laboratory and radiological workup, including total and differential white blood cell count, hemoglobin level, platelets, serum albumin level, C-reactive protein (CRP), blood culture, and chest x-ray [17].

To avoid possible biased results, the study participants were diagnosed and followed by the same team of physicians and radiologists.

### Operational definitions

- Low birth weight was defined by the WHO as weight at birth < 2500 g irrespective of gestational age [18].
- Exclusive breastfeeding was defined as “no other food or drink, not even water, except breast milk (including milk expressed or from a wet nurse) for the first 6 months of life but allows the infant to receive oral rehydration solution, drops, and syrups (vitamins, minerals, and medicines)” [19].
- Artificial feeding (feeding formula) was defined as a breast milk substitute made from a special dried milk powder. Most infant formula is made from cow’s milk, vitamins, and minerals [19].
- Fully vaccinated: Full vaccination includes all children who had obtained compulsory vaccines, each at specific times of their life, as per the WHO vaccination schedule for Egypt [20]. For example, BCG (bacillus Calmette–Guérin vaccine) and OPV0 (oral polio vaccine) at birth, DTwP-Hib-HepB (diphtheria, Pertussis, tetanus, hepatitis B) at 2, 4, 6 months, …etc.
- Crowding was defined as more than 2 persons per room [21].
- Indoor air pollution was assessed by exposure to paternal smoke in addition to subjective assessment of home aeration [21].
- Prolonged hospital stay was defined as hospital stay > 5 days.
- Sepsis was defined as a life-threatening organ dysfunction caused by a dysregulated host response to infection. Sepsis is a systemic inflammatory response syndrome (SIRS) and suspected or confirmed infection [22]. SIRS meets ≥ 2 of the following criteria, 1 of which must be temperature or WBC count:
  - Pyrexia (> 38.5 °C) or hypothermia (< 36 °C).
  - Age-dependent tachycardia or bradycardia.
  - Tachypnea or need for mechanical ventilation.
  - Abnormal WBC count or > 10% immature neutrophils [22].
- Thrombocytopenia was defined as a platelet count ≤ 150 × 10^9^ /L.

The nutritional status of the study subjects was assessed as per the World Health Organization (WHO) child growth standards [23], as follows:

#### Underweight

The participant's weight was plotted against age on a graph for comparison with the standard curve. A low weight-for-age is termed as underweight, defined as a weight-for-age Z-score (WAZ) of less than -2. Severely underweight is classified if WAZ is less than -3 of the WHO (2006) reference values.

#### Wasting

Wasting is defined as a weight-for-height Z-score (WHZ) of less than -2. A Z-score between -2 and -3 is classified as moderate wasting. Severe wasting is classified if WHZ is less than -3 according to the WHO reference standards.

### Stunting

Stunting is a height-for-age Z-score (HAZ) of less than -2. A Z-score between -2 and -3 is considered moderate stunting, and severe stunting is classified if HAZ is less than -3 of the WHO reference standards.

### Study groups

Based on these nutritional parameters the studied participants were classified into two groups: Group (1): Children with pneumonia and with nutritional deficiency considered as “cases”, and group (2): Children with pneumonia and without nutritional deficiency (pneumonia only) considered as “controls”.

For sample size calculation, we used the G-Power version 3.0, with the following parameters:

An estimated rate of 10% of children with pneumonia without nutritional deficiency in the control group, a 90% statistical power to detect an odds ratio (OR) equal to 2.0, with a 95% confidence interval, an alpha error of 5%, and a proportion of 1 case for 1 control. The sample size obtained was 700 patients (350 cases and 350 controls). Due to possible losses, we collected data from 350 cases and 154 controls.

### Ethical considerations

The study has been approved by the Faculty of Medicine Ethical Review Board (IRB No. 17101546). All patients who participated in this study received their medical care as per the hospital management protocols. Written consent was obtained from all participants’ guardians and confidentiality of information was ensured throughout the study.

### Statistical analysis

Quantitative data were statistically described in the form of mean ± SD, and median (IQR) while qualitative data were statistically described in the form of number (percentage). A comparison of quantitative variables was carried out using the Mann–Whitney U test as the data were not normally distributed. For comparing categorical data, Chi-square (χ2) test was used. Fisher Exact test was used instead of Chi-square (χ2) when the expected frequency was less than 5.

A p-value of 0.05 or less was considered to indicate statistical significance. A multivariable regression analysis was performed to analyze the effects of different clinical, demographic, laboratory, radiological, and nutritional parameters on mortality in patients with pneumonia and malnutrition (cases). Statistical analysis was performed using the Statistical Package for Social Science (SPSS) Software (version 24).

## Results

### Demographic and clinical characteristics

**Fig. 1:**
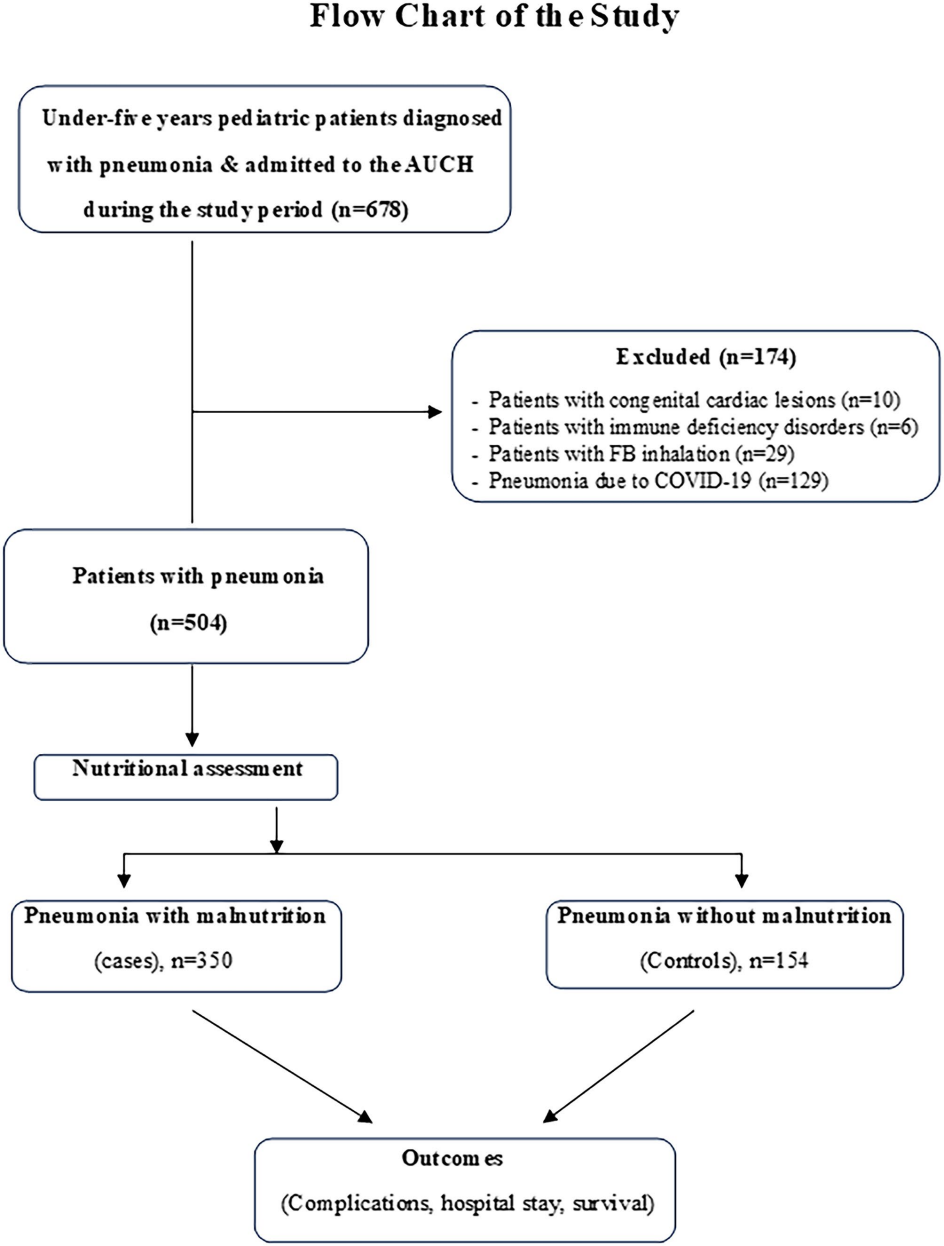
Flow chart of the study

The study included a total of 504 pediatric patients with pneumonia; 350 with nutritional deficiency represented cases versus 154 without nutritional deficiency represented controls. Figure 1 shows the flow chart of the study.

Table 1 details the demographic and clinical characteristics of the study groups. There were significant differences between the cases and control subjects about the age groups and anthropometric parameters (weight, height, and BMI), while there were no significant differences with regaaboutnd residency. There were significant differences between the cases and control subjects with regaabout signs on examination (wheezes, crackles and dimi,nished air entry). Blood and plasma transfusions were significantly higher among cases compared to controls; 34.6% vs 1.9%, and 42.3% vs 1.3%, p < 0.001, respectively (Table 1).

**Table 1:** Demographic and clinical characteristics of the study groups

**Variable**	**Total** **n = 504 (%)**	**Cases** **n = 350 (%)**	**Controls** **n = 154 (%)**	***P*** **value**
**Age groups in years**				0.005
> 1mo-1 yr	233 (46.2)	176 (50.3)	57 (37.0)	
1 – 2y	117 (23.2)	82 (23.4)	35 (22.7)	
2 – 3y	57 (11.3)	39 (11.1)	18 (11.7)	
3 – 4y	55 (10.9)	32 (9.1)	23 (14.9)	
4 – 5y	4 2 (8.3)	21 (6.0)	21 (13.6)	
**Gender**				0.488
Males	267 (53.0)	189 (54)	78 (50.6)	
Females	237 (47.0)	161 (46)	76 (49.4)	
**Residency**				0.696
Urban	216 (42.9)	148 (42.3)	68 (44.2)	
Rural	288 (57.1)	202 (57.7)	86 (55.8)	
**Body weight (Kg)**				< 0.001
Mean ± SD	9.04 ± 4.21	7.78 ± 3.49	11.91 ± 4.31	
**Height**				< 0.001
Mean ± SD	77.06 ± 16.07	73.82 ± 14.70	84.42 ± 16.66	
**BMI (Kg/m**^**2**^**)**				< 0.001
Mean ± SD	14.56 ± 3.23	13.78 ± 3.34	16.34 ± 2.07	
**Feeding**				< 0.001
Exclusive breastfeeding	157 (31.2)	79 (22.6)	78 (50.6)	
Artificial feeding	161 (31.9)	139 (39.7)	22 (14.3)	
Mixed feeding	186 (36.9)	132 (37.7)	54 (35.1)	
**Low birth weight**	54 (10.7)	48 (13.7)	6 (3.9)	0.001
**Compulsory vaccination**	350 (69.4)	217 (62.0)	133 (86.4)	< 0.001
**Crowding index**				< 0.001
< 2	294 (58.3)	185 (52.9)	109 (70.8)	
≥ 2	210 (41.7)	165 (47.1)	45 (29.2)	
**Passive smoking**	262 (52.0)	209 (59.7)	53 (34.4)	< 0.001
**Previous admission**	206 (40.9)	183 (52.3)	23 (14.9)	< 0.001
**Anemia**	319 (63.3)	267 (76.3)	52 (33.8)	< 0.001
**Blood transfusion**	124 (24.6)	121 (34.6)	3 (1.9)	< 0.001
**Plasma transfusion**	150 (29.7)	148 (42.3)	2 (1.3)	< 0.001

*BMI* body mass index (Kg/m2)

### Risk factors for pneumonia

About risk factors for pneumonia, cases had higher rates of artificial and mixed feeding (39.7% and 37.7% vs 14.3% and 35.1%, p < 0.001), low birth weight (13.7% vs 3.9%, p = 0.001), crowding index (47.1% vs 29.2% p < 0.001), and previous hospital admission (52.3% vs 14.9%, p < 0.001), compared to controls, respectively. Cases had lower rates of compulsory vaccination (62.0% vs 86.4%, p < 0.001) and exclusive breastfeeding (22.6% vs 50.6%, p < 0.001), than controls, respectively Table 1.

### Initial laboratory and radiological findings

Cases had significantly higher values of total white blood counts, absolute neutrophilic count (ANC), and C-reactive protein compared to controls, respectively. On the other hand, cases had significantly lower hemoglobin, lymphocytic count, platelets, and albumin values, compared to controls, respectively. Radiological findings were significantly different between the cases and controls. Table 2 details these findings.

**Table 2:** Laboratory & radiological characteristics and outcomes of the study groups^a^

**Variable**	**Cases** **n = 350 (%)**	**Controls** **n = 154 (%)**	***P*** **value**
**Laboratory parameters**^**b**^			
**Hemoglobin** (g/dl) (Normal range 1–23 mo: 10.5–14 2–9 years: 11.5–14.5)	9.54 ± 2.34	11.82 ± 1.87	< 0.001
**Total WBC** (× 10^3^ cells/ml^3^) (Normal range 1–23 mo: 6–17 2–9 years: 4–15.5)	16.74 ± 8.09	14.16 ± 4.88	< 0.001
**Neutrophils** (× 10^3^ cells/ml^3^) (Normal range 54–62% of total WBC)	61.44 ± 17.47	47.93 ± 14.54	< 0.001
**Lymphocytes** (× 10^3^ cells/ml^3^) (Normal range; 25–33% of total WBC)	29.62 ± 16.74	42.08 ± 14.23	< 0.001
**Platelets** (× 10^3^ cells/ml^3^) (Normal range; 150–450)	285.40 ± 207.96	327.28 ± 150.36	< 0.001
**C-reactive protein** (mg/dL) (Normal range; < 0.8)	85.10 ± 66.03	17.49 ± 20.99	< 0.001
**Albumin** (g/dL) (Normal rang**e;** 8 days -1 year: 1.9–4.9 1–3 years: 3.4–5.2 4–19 years: 3.4–5.6)	2.13 ± 0.33	3.99 ± 0.49	< 0.001
**Radiological findings**			
Normal	25 (7.0)	28 (18.0)	< 0.001
Bronchopneumonia	136 (39.0)	74 (48.0)	
Lobar	106 (30.0)	25(16.2)	
Effusion	61 (17.4)	23 (15.0)_	
Aspiration pneumonia	22 (6.6)	4 (2.8)	
**Complications**			
**Heart failure**	93 (26.6)	0 (0)	< 0.001
**Pleural effusion**	61 (17.4)	23 (15.0)	< 0.001
**Myocarditis**	168 (48.0)	0 (0)	< 0.001
**Outcomes**			
**Hospital stay** (> 5 days)	247 (70.6)	29 (18.8)	< 0.001
**Mortality**			< 0.001
Survived	261 (74.6)	154 (100)	
Died	89 (25.4)	0(0)	

*WBC* white blood cells

^a^Laboratory and radiological findings are initial

^b^Ref [17]

### Prevalence of malnutrition among cases

**Fig. 2:**
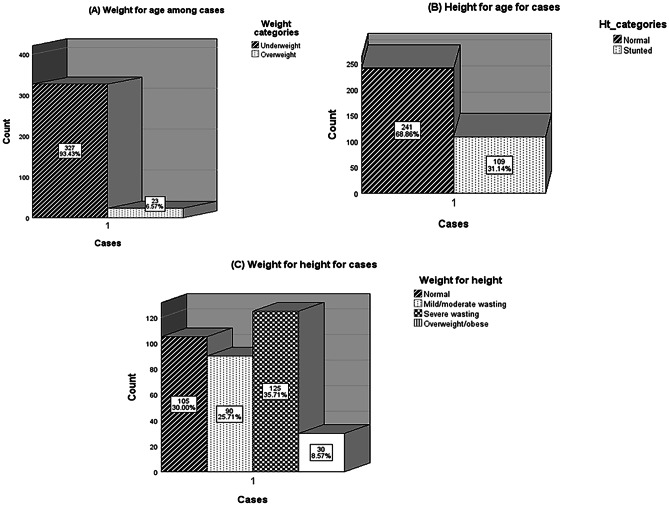
Prevalence of malnutrition among children with pneumonia and malnutrition (cases, n = 350). **A** Weight for age **B** Height for age, and **C** Weight for height

Results showed that (1) For the weight-for-age Z-score (WAZ): the majority of the studied cases (93.4%) were underweight, while 6.6% were overweight. (2) For height-for-age Z-score (HAZ): 31.1% were stunted, while 68.9% had normal HAZ. (3) For weight-for-height Z-score (WHZ): 30.0%, 25.7%, 36%, and 8.3% had normal WHZ, mild/moderate wasting, severe wasting, and overweight/obesity, respectively. Figure 2 shows these data.

It was shown that younger aged patients (< 1 year) were more likely to be stunted (P = 0.002) and wasted (P < 0.001). The weight-for-age Z-score was comparable among different age groups. Male patients were more likely to be wasted compared to females (68.3% vs. 54.0% P = 0.022). About the type of feeding, patients who fed on artificial or mixed feeding were more likely to be stunted (P < 0.001) and wasted (P = 0.036) compared to children who had exclusive breastfeeding.

### Outcomes (complications, hospital stay, and survival)

The cases group developed significantly higher rates of complications (heart failure and pleural effusions), and they had a significantly longer duration of hospitalization (70.6% vs 18.8%, p < 0.001), compared to controls, respectively. During the hospital stay, 89/350 (25.4%) of the cases died versus none of the controls, p = 0.001, respectively. Table 2 shows these details.

### The group of cases: survivors versus non-survivors

Comparisons between cases who survived (n = 261) and those who did not (n = 89), by univariable analysis revealed significant results.

There were significant differences between survivors and non-survivors with regard to some clinicodemographic factors, laboratory parameters, and anthropometric parameters. There were significant differences between survivors and non-survivors about feeding, vaccination status, presence of previous admission, heart failure, sepsis, thrombocytopenia, anemia, and positive CRP. Also, there were significant differences about prolonged hospital stay (> 5 days), blood and plasma transfusion, and the presence of stunting. Table 3 details these results.

**Table 3:** Survivors versus non-survivors among cases, n = 350 (Univariate analysis)

**Variable**	**Survivors** **N = 261 (74.5%)**	**Non-survivors** **N = 89 (25.5%)**	***P-*** **value**
Age groups			0.076
1m-1y	120 (46.0)	56 (63.0)	
1-2y	64 (24.5)	18 (20.0)	
2-3y	33 (12.6)	6 (6.7)	
3-4y	27 (10.3)	5 (5.6)	
4-5y	17 (6.6)	4 (4.7)	
Gender (male)	145(55.6)	44 (49.4)	0.317
Feeding			< 0.001
Exclusive Breastfeeding	73 (28.0)	6 (6.0)	
Artificial feeding	91(34.8)	48 (54.0)	
Mixed feeding	97 (37.2)	35 (40.0)	
Low birth weight (yes)	41(15.7)	7 (7.8)	0.074
Crowdening (≥ 2)	120 (46)	45 (50.5)	0.455
Compulsory vaccination (no)	89 (34)	44 (49.4)	0.011
Passive smoking (yes)	151(57.8)	58 (65.2)	0.225
Previous admission (yes)	122 (46.7)	61(68.5)	< 0.001
Heart failure	46 (17.6)	47 (52.8)	< 0.001
Pleural effusion	30 (11.5)	13 (14.6)	0.440
Sepsis (yes)	108 (41.3)	81(91.0)	< 0.001
Hospital stay (> 5days)	165 (63.2)	82 (92.1)	< 0.001
Thrombocytopenia (yes)	60 (23.0)	67 (75.2)	< 0.001
CRP positive (> 5)	230 (88.0)	87 (97.7)	0.017
Anemia (yes)	182 (69.7)	85 (95.5)	< 0.001
Leukocytosis (yes)	199 (76.2)	62 (69.6)	0.486
Blood transfusion (yes)	57(21.8)	64 (72.0)	< 0.001
Plasma transfusion (yes)	76 (29.0)	72 (81.0)	< 0.001
Underweight (yes)	239 (91.5)	88 (98.8)	0.059
Stunted (yes)	73 (28.0)	36 (40.4)	0.029
Weight for height z score	159 (60.9)	57 (64.0)	0.146

### Predictors of mortality

To analyze the predictors of mortality among children with pneumonia and malnutrition, the multivariable regression analysis revealed that lack of compulsory vaccination, presence of sepsis, and blood transfusion (OR 2.874, 95% CI 0.048 – 2.988, p = 0.004, 2.627, 0.040 – 2.677, p = 0.009, and 4.108, 0.134 – 3.381, p < 0.001, respectively) were significant independent predictors for mortality. Table 4 shows these results.

**Table 4:** Multivariable logistic regression analysis

**Variable**	**Odds Ratio (95% CI)**	***P*** **value**
Feeding	0.425 (0.001 – 0.533)	0.613
Compulsory vaccination	2.874 (0.048 – 2.988)	0.004
Previous admission	1.400 (0.028 – 1.452)	0.162
Heart failure	0.588 (0.008 – 0.666)	0.480
Anemia	0.112 (0.00 – 0.322)	0.821
Sepsis	2.627 (0.040 – 2.677)	0.009
Hospital stay	0.320 (0.002 – 0.455)	0.755
Blood transfusion	4.108 (0.134 – 3.381)	< 0.001
Plasma transfusion	1.367 (0.019 – 1.122)	0.173
Underweight	0.988 ( 0.015 – 0.986)	0.251
Stunted	0.622 ( 0.010 – 0.633)	0.484

## Discussion

The current study was carried out to address the prevalence and impact of malnutrition on under-five years children with pneumonia, admitted to AUCH, over one year. It included 504 pediatric patients with pneumonia; 350 cases with, and 154 controls without nutritional deficiency, respectively.

Notably, the current study enrolled large numbers of both cases and controls, which gives the results their robustness, significance, and clinical implications in daily practice. Risk factors for pneumonia include malnutrition, indoor air pollution, high population density in the house (overcrowding), and the presence of co-morbidities [1, 2, 7–10].

Malnourished children are at a greater risk of developing pneumonia due to their weakened immune systems, decreased ability to fight off infections, and impaired respiratory function [24].

Malnutrition contributes to the severity and frequency of pneumonia cases in several ways. [12, 13, 21, 24] Firstly, it impairs the body's defense mechanisms, reducing the ability to combat pathogens effectively. Secondly, malnourished children may have reduced muscle mass, including the respiratory muscles, which can affect breathing and clearance of mucus from the lungs. Thirdly, malnutrition can lead to deficiencies in essential nutrients such as vitamin A, zinc, and iron, which are necessary for a robust immune response [12, 13, 21, 24].

In the current study, artificial and mixed feeding, low birth weight, non-adherence to compulsory vaccination, higher crowding index, passive smoking, and previous hospital admission, have been identified as risk factors for patients with pneumonia and malnutrition. This is in agreement with observations by Sutriana et al. [25] who observed that no or non-exclusive breastfeeding, incomplete basic immunizations, indoor air pollution, a history of low birth weight, and severe malnutrition were risk factors for childhood pneumonia [25]. Our results are in favor of the recommendation that exclusive breastfeeding until the age of 6 months and receiving complete basic vaccination are protective factors for the development of pediatric pneumonia [19, 25, 26].

The laboratory findings of the current study were interesting. Malnourished children with pneumonia had significantly higher values of leukocytes, absolute neutrophilic count, and C-reactive protein, and lower values of hemoglobin, lymphocytic count, platelets, and albumin, compared to controls, respectively. These findings are in agreement with previous studies [7–11]. Serum procalcitonin (PCT) and CRP have been shown to predict positive blood culture among children with severe pneumonia, though that does not rule out infection in malnourished children [10]. Shahrin et al. [10], found that several biomarkers at the time of presentation were able to predict 30-day mortality of children with severe malnutrition and severe pneumonia, including PCT, CRP, and polymorphonuclear (PMNL) percentage. However, after adjusting for potential confounders only a higher PMNL percentage remained to be associated with deaths and may be used for predicting these deaths [10]. We agree with reports of previous studies that showed that combining biomarkers levels with clinical predictors improves prognostication of children with pneumonia, and sepsis [27].

In the current study, we observed a high prevalence of malnutrition among under-five children with pneumonia. Among 350 patients with pneumonia, 93.4%, 31.1%, and 61.7% suffered from underweight, stunting, and wasting, respectively. This prevalence rate was worse than that documented by the recent Egyptian study of El-Koofy et al. [13], which found that malnutrition (underweight, wasting, or stunting) was detected in 12.4% of all children with upper and lower respiratory tract infections (RTIs), with 4.9% of them having stunting [13]. Remarkably, malnutrition was closely related to the type of feeding. Our results showed that patients who had artificial or mixed feeding were more likely to be stunted and wasted, compared to those who had exclusive breastfeeding.

The protective effect of human milk against respiratory infection is attributed to its numerous immunobiological components [28]. The immunoglobulins found in human milk include IgA, secretory IgA (SIgA), IgM, secretory IgM (SIgM), and IgG, with SIgA playing a central role in its defense against infectious disease [29]. Cytokines are secreted proteins found in human milk that have an important role in the development of the infant’s immune system through their anti-inflammatory and immunosuppressive properties [29].

The combination of pneumonia and malnutrition has an additive adverse effect on child morbidity. It has been reported in developing countries that severely underweight and moderately wasted children with pneumonia were at 6.4- and 4.2 times higher risk of mortality, compared to the children without pneumonia, respectively [30]. In their recent meta-analysis, Kirolos et al. [12] concluded that The risk of death from childhood pneumonia dramatically increases with malnutrition severity. This risk has remained high in recent years with an estimated over half of in-hospital pneumonia deaths attributable to child malnutrition [12].

Our results had shown that there were significant differences between survivors and non-survivors with regard to feeding, vaccination status, presence of previous admission, heart failure, sepsis, thrombocytopenia, anemia, positive CRP, prolonged hospital stay (> 5 days), blood and plasma transfusion, and the presence of stunting. Lack of compulsory vaccination, presence of sepsis, and blood transfusion were significant independent predictors of mortality in malnourished children with pneumonia. In the study of Shahrin and coworkers [10]; female sex, severe stunting < _4HAZ, and higher PMNL percentage were independently associated with 30-day mortality in a cohort of severely malnourished children with pneumonia. Another study by Chisti et al., [11] has identified hypoxemia, clinical dehydration, and abdominal distension as the independent predictors of death in children with severe acute malnutrition and pneumonia.

Our results imply that early identification and prompt management of these clinically recognizable predictors of death may help reduce deaths in such populations. Early recognition and management of those risk factors could improve outcomes of under-five children with pneumonia and malnutrition, particularly in resource-limited areas and developing countries. Prevention and treatment of child malnutrition must therefore be prioritized by healthcare policymakers to maintain progress on reducing pneumonia.

## Strengths and limitations

The current study has some points of strength. It is a prospective study that enrolled a large number of both cases and controls in a large tertiary center. Results are representatives of larger population ones. On the other hand, our study has some limitations. First, the limitation of a single-center experience is encountered. Second, Low birth weight included in the study could be associated with biased results. Low birthweight babies will have time to catch growth based on their condition. Further, multicentred studies are warranted.

## Conclusions

Malnutrition has a high prevalence in under-five children with pneumonia in our locality. It has adverse effects on the outcomes and in-hospital mortality of those children. Lack of compulsory vaccination, the presence of sepsis, and blood transfusion were significant independent predictors of mortality in malnourished children with pneumonia. Early recognition and management of those predictors could improve outcomes of under-five children with pneumonia and malnutrition, particularly in resource-limited areas. Further larger multicenter studies are warranted.

## Data Availability

The datasets used and/or analyzed during the current study are available from the corresponding author upon reasonable request.
